# Optically Transparent Dual-Ring Resonant Frequency Selective Surface Based on ITO Film for Sub-6 GHz Indoor Communication

**DOI:** 10.3390/mi17060656

**Published:** 2026-05-26

**Authors:** Yujuan Wei, Ruichao Zhu, Shulei Zhang, Fangyuan Qi, Ya Fan, Zhaotang Liu

**Affiliations:** 1 Shaanxi Key Laboratory of Artificially-Structured Functional Materials and Devices, Air Force Engineering University, Xi’an 710051, China; weiyujuan082@163.com (Y.W.); zhuruichao1996@163.com (R.Z.); 18365785171@163.com (S.Z.); qfyyyyy678@163.com (F.Q.); 2 Air Force Early Warning Academy, Wuhan 430019, China; 3 Suzhou Laboratory, Suzhou 215006, China

**Keywords:** metasurface, frequency-selective surface (FSS), indium tin oxide (ITO), electromagnetic interference (EMI), optical transparency, sub-6 GHz

## Abstract

With the rapid development of wireless communications, electromagnetic interference (EMI) in complex environments has become a critical factor affecting communication quality. Addressing the EMI issues caused by multi-band coexistence in indoor scenarios, traditional metallic resonant structures, while effective in filtering, often compromise optical transparency due to light blockage. To resolve this trade-off, this paper proposes a dual-ring resonant frequency-selective surface (FSS) based on Indium Tin Oxide (ITO) films. This design aims to achieve efficient transmission in specific C-band frequencies and suppress out-of-band interference, realizing excellent optical transmittance while ensuring electromagnetic shielding effectiveness. The designed metasurface targets a passband of 5.35–5.40 GHz for sub-6 GHz indoor communications. Experimental results confirm superior transmission in this range and significant out-of-band suppression. Furthermore, featuring high optical transparency, the structure can be directly integrated onto glass surfaces. It is not only suitable for optically transparent devices but also provides a compact passive solution for anti-EMI applications in smart buildings and sub-6 GHz indoor communications.

## 1. Introduction

In recent years, with the rapid development of wireless communication technology, indoor communication scenarios have imposed more stringent requirements on signal quality. However, multi-band interference is prevalent in complex electromagnetic environments, which severely restricts the stable operation of communication systems. Therefore, suppressing electromagnetic interference (EMI) and achieving effective transmission of electromagnetic waves in specific frequency bands have become key to guaranteeing indoor communication performance.

Electromagnetic interference (EMI) originates from electromagnetic waves generated by the operation of electronic devices or systems. It affects the performance of surrounding devices, systems, or even the source itself through radiation or conduction. In complex indoor environments, the impact of EMI on signal quality cannot be overlooked, which imposes higher requirements on EMI suppression effectiveness [1,2]. Metasurfaces have garnered widespread attention in the communication field due to their excellent performance in frequency-selective filtering. To achieve more efficient transmission of useful signals and suppress out-of-band interference, numerous frequency-selective metasurfaces have been proposed for EMI suppression [1,2,3,4,5,6,7,8]. As interference sources multiply, simple band-pass or band-stop capabilities are no longer sufficient; consequently, metasurface designs must possess characteristics of high selectivity and wide stopbands. Ref. [3] proposes a multi-layer cascaded structure that achieves rapid band edges in the Ka-band and interference shielding in the V-band. In modern smart buildings and indoor communication scenarios, EMI suppression requires solutions that offer both optical transparency and filtering capabilities. In this context, a Glass-Penetrating Transparent Surface (GPTS) for 5G indoor communication was proposed and verified in [4]. Its core is a single-layer structure based on a frequency-selective surface (FSS), which realizes transmission enhancement and out-of-band suppression for millimeter-wave signals while maintaining the optical transparency of the glass. Furthermore, a metallic mesh design employing a cross structure has been proposed. By adjusting the line width of the mesh, optical transmittance can be significantly improved without compromising transmission performance [5]. However, in practical applications, the variability of signal incident angles necessitates superior angle stability, while the trend toward device miniaturization requires more compact structural designs. Addressing EMI shielding for 5G millimeter-wave bands, a dual-polarized conformal design was proposed with a period of only 0.26 $\lambda_{0}$, supporting incident angles up to 60° [6]. The single-layer planar structure presented in [7], aimed at shielding WLAN hotspots, achieves miniaturization through an interlocking ring design. With a unit cell size of merely 0.05 $\lambda_{0}$, it maintains stable shielding effectiveness even at incident angles as high as 75°. Furthermore, Ref. [6] proposes a metasurface filter antenna based on characteristic mode analysis that operates without additional filtering circuits. To overcome the limitations of single-function materials, Ref. [8] designed a multifunctional metasurface integrating optical, thermal, and electromagnetic functions. By combining randomized metallic meshes with Indium Tin Oxide (ITO), it simultaneously achieves high visible light transmittance, robust shielding characteristics, and thermal insulation in the infrared band. Although existing research has focused on EMI suppression technologies, the suppression of interference in scenarios where multiple frequency bands coexist within indoor communications remains an area requiring further exploration [9,10,11,12,13].

**Figure 1:**
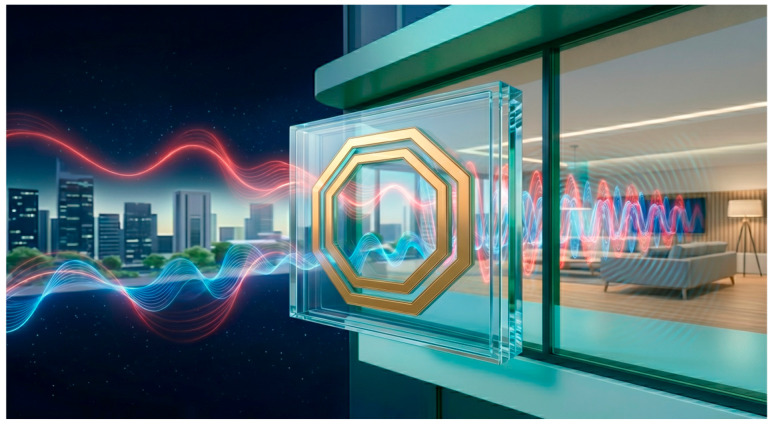
The functional schematic.

This paper proposes an ITO-based metasurface utilizing a double-ring resonant unit cell, where the top and bottom layers consist of perfectly symmetric octagonal double-ring structures. Based on this proposed unit, a 19 × 19 transmission array was designed, fabricated, and tested in the C-band using a horn antenna as the feed source. Experimental results demonstrate that the designed metasurface ensures favorable signal transmission within the 5.35–5.40 GHz band while effectively filtering out-of-band interference signals. Validation via a communication system confirms successful in-band data packet transmission, exhibiting promising application potential. Furthermore, characterized by high optical transparency, this metasurface can be applied to glass substrates to suppress out-of-band interference, laying a foundation for applications in optically transparent devices. Existing research on transparent electromagnetic windows based on frequency-selective surfaces (FSSs) has primarily focused on high-frequency millimeter-wave bands, with insufficient attention paid to frequencies below 6 GHz. Moreover, conventional designs often employ metallic meshes or multi-layer structures [14,15,16,17,18], which suffer from limited optical transparency [19,20]. In comparison, the proposed metasurface features the advantages of a simple structure and ease of fabrication. The transparent FSS designed in this paper focuses on sub-6 GHz, combining high transparency with strong in-band transmission and out-of-band suppression capabilities. And its communication effectiveness is verified through practical data packet transmission tests. The functional schematic is shown in Figure 1, demonstrating its unique value in electromagnetic interference suppression and indoor communication enhancement for transparent architectural glass.

The remainder of this paper is organized as follows. Section 2 presents the proposed unit cell and array configurations, along with the CST simulation results. Section 3 details the measurements of the fabricated prototype and discusses the results, followed by validation within a communication system. Finally, conclusions are drawn in Section 4.

## 2. Unit Cell Design and Simulation

### 2.1. Dual-Ring Resonator Design

**Figure 2:**
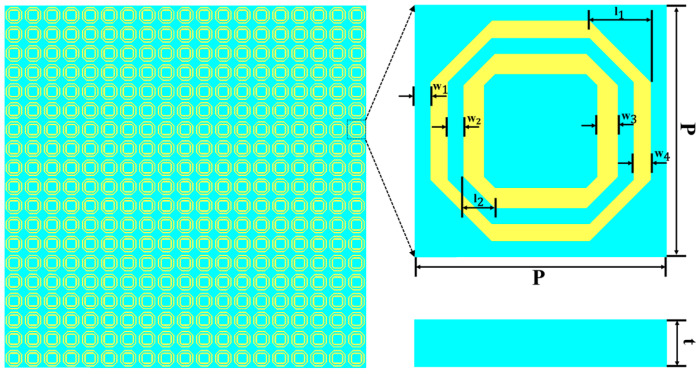
Schematic of the array and unit cell.

Figure 2 illustrates the proposed unit cell structure and the array configuration. The unit cell consists of a quartz substrate coated with two identical ITO films on its top and bottom surfaces. The structure features a full octagonal dual-ring geometry with concentric inner and outer rings, exhibiting up–down symmetry. Operating in the C-band, the unit cell has a periodicity of P = 16 mm. The substrate thickness is t = 3 mm, with a relative permittivity of 3 and a loss tangent of 0.06. As depicted in Figure 2, $w_{1}$ denotes the width between the outer ring and the unit cell boundary, $w_{2}$ represents the gap between the outer and inner rings, and $w_{3}$ and $w_{4}$ correspond to the ring widths of the inner and outer rings, respectively.

To validate the proposed design, full-wave electromagnetic simulations were performed using CST Microwave Studio 2022, which is based on the Finite Integration Technique (FIT). In the simulation setup, unit cell boundary conditions were applied in the x and y directions to mimic an infinite periodic array. The z direction was set as an open boundary with Perfectly Matched Layers (PMLs) to absorb reflected waves, and floquet ports were configured at $Z_{\min}$ and $Z_{\max}$ for two modes. A plane electromagnetic wave was incident along the negative z-axis. Fixed-frequency monitors were employed to observe the surface electric field and current distributions, with the frequency domain solver sweeping from 4 to 7 GHz.

**Figure 3:**
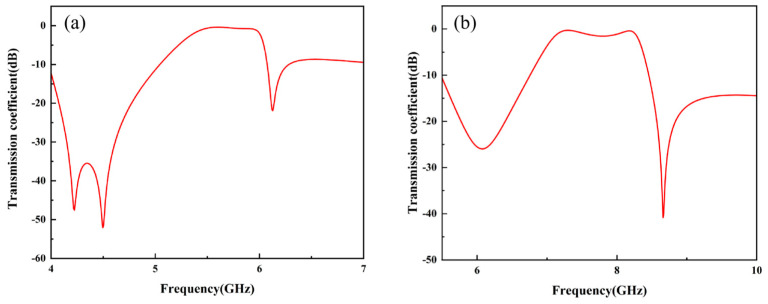
Simulated transmission coefficient (S21) of the unit cell: (**a**) simulation results under ideal conditions; (**b**) simulation results with air layers.

To analyze the performance, the simulation results shown in Figure 3 were examined. The two resonant dips in the $S_{21}$ curve exhibit typical asymmetric characteristics, revealing the contribution of the Fano resonance effect. The inner ring produces the high-frequency resonance, while the outer ring is responsible for the low-frequency resonance. Given that the inner ring is wider ($w_{3}$ = 1.3 mm) compared to the outer ring ($w_{4}$ = 1.1 mm), it possesses a lower equivalent resistance and a higher Q-factor, causing a steeper decline in the high-frequency resonance peak. In contrast, the narrower outer ring has a higher equivalent resistance and a lower Q-factor, leading to a more gradual slope on the low-frequency resonance peak. Under the ideal simulation setup (glass $\varepsilon_{r}$ = 3.79, no PET layer, perfect ITO adhesion), the metasurface exhibits excellent transmission in the 5.5–6.0 GHz band, as shown in Figure 3a. However, as will be discussed in Section 3, practical material deviations shift this passband to 5.35–5.40 GHz. The simulation serves as a proof of concept and parametric study, while the measured results represent the actual performance. Compared to the ideal case where the ITO film is perfectly bonded to the glass, the transmission coefficient decreases when there is an air gap between the ITO and the glass. The presence of the air layer causes impedance mismatch, thereby affecting the performance of the metasurface, as shown in Figure 3b.

**Figure 4:**
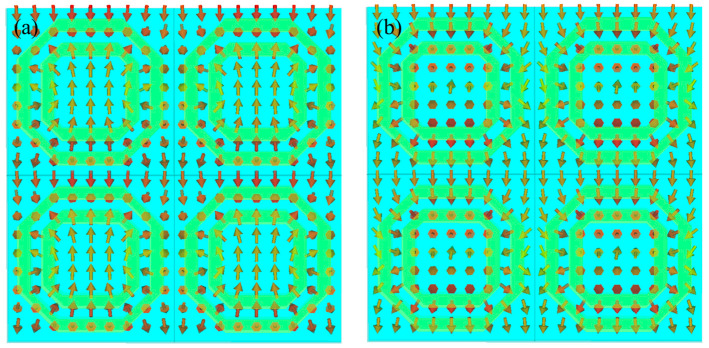
Electric field distribution: (**a**) electric field distribution at 4.5 GHz; (**b**) electric field distribution at 6.1 GHz.

To further elucidate the mechanism, the electric field distributions of the unit cell were investigated, as shown in Figure 4a,b. These figures demonstrate the mutual coupling between the inner and outer rings. By observing the field distributions at 4.5 GHz and 6.1 GHz, reversed electric field lines can be seen, revealing a current phase inversion. It is worth noting that the effective inductance (L) arises from the conductor itself, while the effective capacitance (C) is attributed to the gap between the outer and inner rings.

### 2.2. Resonance Mechanism Analysis

When electromagnetic waves are incident, the induced currents in the ring structures form closed loops, generating an electromagnetic response analogous to a magnetic dipole, with the magnetic moment oriented along the ring axis. The electromagnetic coupling between the two rings enhances this magnetic dipole response, leading to a stronger resonant effect. When the frequency of the incident wave matches the natural resonant frequency of the structure, the magnetic dipole response reaches its maximum, resulting in magnetic resonance, which manifests as a transmission valley.

**Figure 5:**
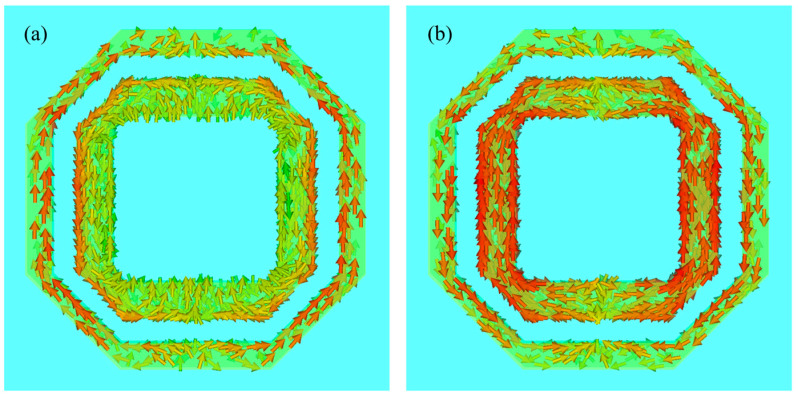
Current distribution: (**a**) current distribution at 4.5 GHz; (**b**) current distribution at 6.1 GHz.

As shown in Figure 5a, observing the current distribution at 4.5 GHz reveals that the inner ring current follows a magnetic dipole field line distribution, while the outer ring current flows in the opposite direction. In Figure 5b, the inner and outer ring currents also flow in opposite directions, but their phase is reversed compared to that at 4.5 GHz. This is attributed to the two magnetic dipole resonance modes generated by the inner and outer rings. The smaller inner ring possesses a smaller equivalent inductance, which results in a higher resonant frequency. In contrast, the larger outer ring has a larger equivalent inductance, and therefore governs the lower resonant frequency.

### 2.3. Parameter Sweep and Optimization

**Figure 6:**
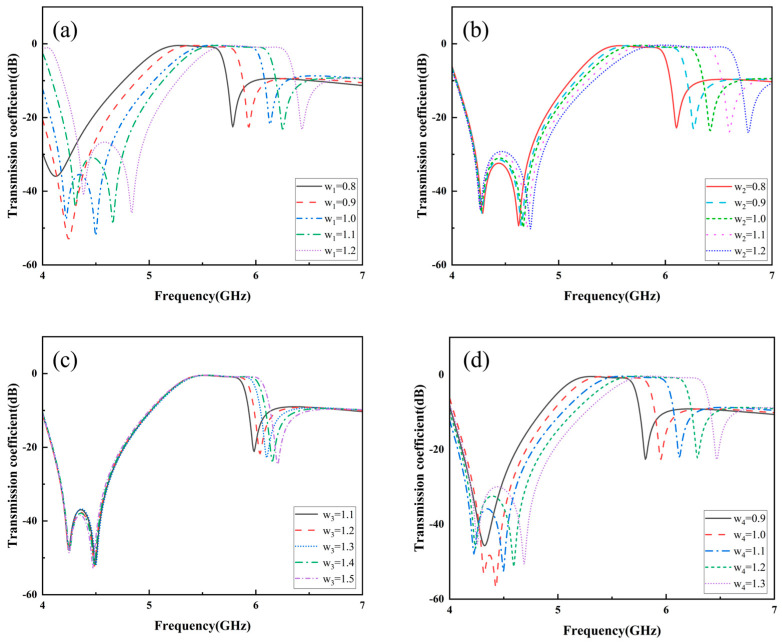
Parameter scanning: (**a**) parametric sweep of $w_{1}$; (**b**) parametric sweep of $w_{2}$; (**c**) parametric sweep of $w_{3}$; (**d**) parametric sweep of $w_{4}$.

Geometric parameters such as the ring radius, width, ring spacing, and substrate thickness directly influence the resonant frequency. To achieve optimal unit cell performance, a parametric sweep was conducted on key dimensional parameters. The inner and outer ring widths and the gap size were adjusted individually to optimize the dimensions. When increasing the distance $w_{1}$ between the outer ring and the unit cell boundary, as shown in Figure 6a, both the high- and low-frequency resonant points shifted toward higher frequencies. Conversely, increasing the dual-ring spacing $w_{2}$, as depicted in Figure 6b, caused the high-frequency resonance to shift upward, while the low-frequency resonance remained virtually unchanged.

Ultimately, the geometric parameters of the finalized unit cell are listed in Table 1. Figure 6c illustrates the variation in the inner ring width $w_{3}$ from 1.1 to 1.5 mm; as the inner ring width increases, the high-frequency resonant point shifts toward a higher frequency, with the low-frequency resonance remaining basically constant. Figure 6d demonstrates the variation in transmission amplitude as the outer ring width $w_{4}$ changes from 0.9 to 1.3 mm. It can be observed that, as the outer ring width increases, both high- and low-frequency resonant points shift toward higher frequencies.

### 2.4. Angular Stability Analysis

**Figure 7:**
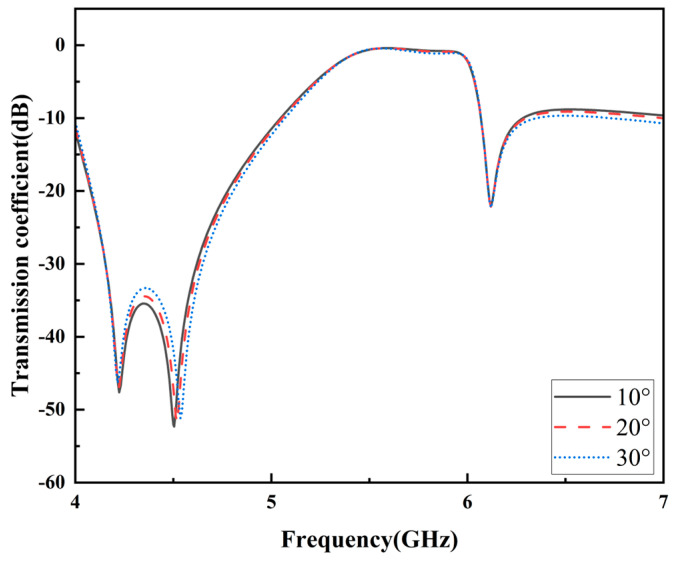
Simulated transmission coefficient (S21) of the proposed FSS under oblique incidence, with incident angles of 10°, 20° and 30°.

In practical application scenarios, electromagnetic waves do not usually transmit through glass at strictly normal incidence; therefore, the angular stability of the metasurface is crucial. Figure 7 presents the simulated transmission coefficients under three typical incident angles. The results show that the transmission coefficient remains almost unchanged at different incident angles, verifying that the designed metasurface possesses excellent angular stability, which is sufficient for typical indoor communication scenarios.

## 3. Measurements

**Figure 8:**
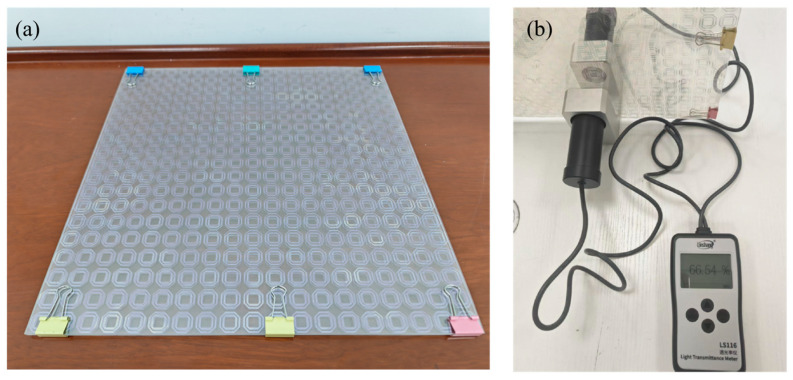
The processed sample and the transmittance test: (**a**) the processed sample; (**b**) image of transmittance test.

Based on the design analysis, a metasurface sample with dimensions of 304 × 304 mm^2^ was precisely fabricated using standard PCB technology, as shown in Figure 8a. The ITO films on both sides are deposited on a PET substrate with a total thickness of 0.125 mm, and the sheet resistance of the ITO is 2.5 Ω/sq.

As shown in Figure 8b, the transmittance tester LS117 (Keysight Technologies, Inc., Santa Rosa, CA, USA) measured the sample’s transmittance at 66.54%, which demonstrates its favorable optical transparency.

### 3.1. Transmission Coefficient

**Figure 9:**
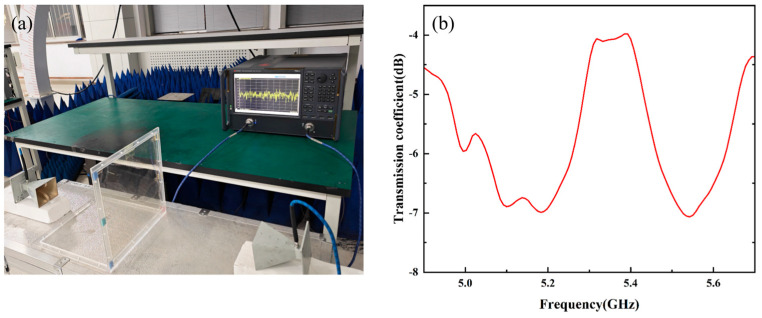
Experimental and measurement results: (**a**) experimental setup; (**b**) experimental results.

To meet the testing requirements, the flexible ITO films and the glass substrate were stably assembled into an integrated test unit, as depicted in Figure 9a, ensuring no relative displacement at the material interface during the measurement.

The test system, centered around a Vector Network Analyzer (VNA), employed standard gain horn antennas operating in the 2–18 GHz range connected to Port 1 and Port 2, respectively. These antennas were symmetrically arranged on both sides of the sample to constitute a free-space transmission test setup. The calibrated system scanned the transmission coefficient of the sample in the 4–7 GHz range. The measured $S_{21}$ curve is presented in Figure 9b.

The data indicate that, within the target frequency band of 5.35–5.40 GHz, the sample exhibits significant passband transmission characteristics, while showing obvious suppression of out-of-band signals. It is worth noting that the measured insertion loss is higher than the simulated curve. This performance degradation is primarily attributed to the imperfect adhesion between the ITO films and the glass substrate. The presence of air gaps alters the effective dielectric environment between the metasurface and free space, thereby leading to the discrepancy between the measured results and the ideal simulation model. Additionally, as a non-ideal conductor, the sheet resistance of ITO induces Joule heating from surface currents. The induced current peaks when the incident EM wave frequency nears the resonance of the unit cell, resulting in a marked rise in insertion loss. However, these test results clearly verify that the designed metasurface possesses band-pass filtering characteristics within the target band, providing a reliable experimental basis for subsequent engineering applications.

### 3.2. Communication System Testing

**Figure 10:**
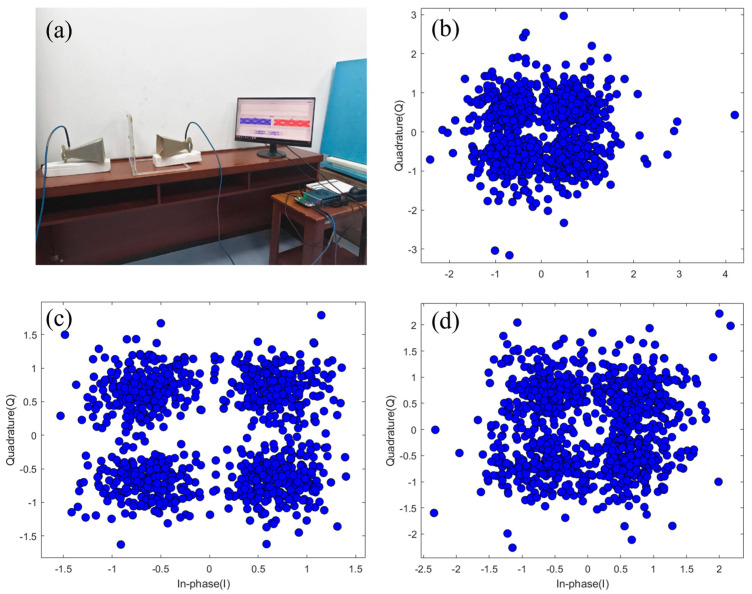
Schematic diagram of the communication system test: (**a**) experimental setup; (**b**) constellation diagram at 5.0 GHz; (**c**) constellation diagram at 5.3 GHz; (**d**) constellation diagram at 5.6 GHz.

Finally, to comprehensively evaluate the practical transmission performance of the designed metasurface, a broadband wireless communication test system was established to characterize the sample, as shown in Figure 10a. The setup process involved the following steps: selecting horn antennas operating in the 2–18 GHz range as the transceiver units, assembling the transceivers and signal processing modules, configuring the modulation/demodulation parameters and communication protocols, and finally placing the designed metasurface between the two horn antennas. During the test, a QPSK modulated signal was injected via a signal generator. A spectrum analyzer was used to monitor the spectral purity of the transmitted signal, while a vector signal analyzer was employed to synchronously collect data at the receiving end. Constellation diagrams at different frequency points were obtained after demodulation.

The test results are presented in Figure 10b–d, showing the QPSK constellation diagrams for data transmission at 5.0 GHz, 5.3 GHz, and 5.6 GHz, respectively. Measured at 5.3 GHz, which is slightly below the measured passband center, the constellation diagram exhibits an ideal distribution with four clusters of symbol points highly converging and clear, distinguishable boundaries. This confirms that the metasurface achieves low-loss, high-fidelity signal transmission at this frequency, which is in good agreement with the simulated passband characteristics. In contrast, the severe diffusion of symbol points and the overlapping blur between clusters observed at 5.0 GHz and 5.6 GHz demonstrate the expected efficient suppression capability against out-of-band interference signals.

## 4. Conclusions

This study proposes a metasurface with frequency-selective filtering capabilities. By systematically analyzing the electric field and current distributions, the coupling characteristics of resonance modes and the phase transition process are revealed. To verify its communication effectiveness, the metasurface was tested within a communication system. It achieved efficient transmission at 5.3 GHz while exhibiting significant suppression at 5.0 GHz and 5.6 GHz, thereby validating its narrow band-pass filtering characteristics and high frequency selectivity. Through structural optimization, this design effectively regulates electromagnetic resonance, providing a high-performance, compact passive filtering solution for band isolation and interference suppression in communication systems. It holds application potential in fields sub-6 GHz, radar, and the Internet of Things (IoT). In addition, this dual-ring ITO metasurface lays a solid theoretical and practical foundation for scaling up to large-area smart windows or architectural glass. The key lies in overcoming challenges related to large-area ITO uniformity, double-sided alignment accuracy, and pattern consistency. Adopting R2R sputtering combined with photolithography, or high-precision mask photolithography, represents the most engineering-feasible pathway.

## Figures and Tables

**Table 1:** Geometric parameters.

P	$l_{1}$	$l_{2}$	$w_{1}$	$w_{2}$	$w_{3}$	$w_{4}$	t
16	3.9	2	1	1	1.3	1.1	3

## Data Availability

The original contributions presented in this study are included in the article. Further inquiries can be directed to the corresponding author.

## Abbreviations

The following abbreviations are used in this manuscript:

EMI	Electromagnetic Interference
GPTS	Glass-Penetrating Transparent Surface
FSS	Frequency-Selective Surface
ITO	Indium Tin Oxide
IoT	Internet of Things
